# Gender differences and psychosocial stress in upper respiratory tract infections: insights from healthy and hematological cancer cohorts

**DOI:** 10.1186/s12889-026-26732-7

**Published:** 2026-05-30

**Authors:** Maria Madeleine Rüthrich, Leonie Ascone, Jakob Hammersen, Inken Hilgendorf, Jana Kalkreuth, Stephanie Kurze, Eva Maria Peters, Tobias Rachow, Jenny Rosendahl, Marie von Lilienfeld-Toal

**Affiliations:** 1 Department of Nephrology and Medical Intensive Care, Charité Universitätsmedizin Berlin, Berlin, Germany; 2 Department of Psychiatry and Psychotherapy, Neuroplasticity Working Group, University Medical Center Hamburg-Eppendorf, Hamburg, Germany; 3 Institute for Diversity Medicine, Medical Faculty, Ruhr-University Bochum, MB 5/131, Universitätsstr. 150, 44801 Bochum, Germany; 4 Department of Internal Medicine II, Hematology and Medical Oncology, University Hospital Jena, Jena, Germany; 5 Psychoneuroimmunology Laboratory, Department of Psychosomatic Medicine and Psychotherapy, Justus-Liebig University Giessen, Giessen, Germany; 6 Department of Psychosomatic Medicine and Psychotherapy, Universitätsmedizin- Charité Berlin, Berlin, Germany; 7 Institute of Psychosocial Medicine, Psychotherapy and Psychooncology, Friedrich- Schiller-University Jena, Jena, Germany; 8 Leibniz Institute for Natural Product Research and Infection Biology, Hans-Knöll Institute, Jena, Germany

**Keywords:** Gender disparities, Stress, Upper respiratory tract infections (URTIs), Stem cell transplantation, Psychosocial factors, Health behavior, Cancer

## Abstract

**Background:**

Upper respiratory tract infections (URTIs) are shaped by sex and gender, with stress potentially playing a critical role. However, the interplay remains poorly understood. This study examined whether women experience higher stress than men, contributing to increased URTI susceptibility and symptom burden.

**Methods:**

Our monocentric, prospective study included 273 healthy individuals (cohort H) and 194 stem-cell recipients (cohort P), enrolled between 11/2013 and 05/2017. Participants were aged ≥ 18 years. Data included demographics, household composition, smoking behavior, cancer-related characteristics, URTI-symptoms, and perceived stress (measured via 4-item perceived stress scale, PSS-4). Participants were categorized by biological sex, assuming alignment with gender.

**Results:**

In cohort H (50% women, mostly ≤ 30 years), women reported more moderate/severe URTI-symptoms than men (57% vs. 36%, *p* < 0.001, φ = 0.210) and higher PSS-4 scores [t(271) = 2.84, *p* < 0.001, d = 0.636]. In women, stress modestly correlated with symptom burden (*r* = 0.15, *p* = 0.04). Sex and age were significant predictors of stress. In cohort P (40.5% women, mostly ≥ 30 years), men reported more URTI symptoms (83% vs. 68%, *p* = 0.021), while PSS-4 did not differ by sex. Stress correlated with symptom burden (*r* = 0.293, *p* < 0.001), particularly in women (*r* = 0.385, *p* < 0.001). Symptom burden was the only independent predictor of stress.

**Conclusion:**

The relationship between sex, stress, and URTI burden varies by health status. Healthy women, especially younger adults, experience higher stress and more severe symptoms, though stress only modestly relates to symptom burden. In immunocompromised patients, stress strongly reflects symptom severity, particularly in women, with minimal sex differences in stress. These findings highlight a complex interplay between sex, psychosocial stress, and infection outcomes, emphasizing the need for interventions addressing both biological and psychosocial determinants, particularly in vulnerable populations.

**Trial registration:**

Registry: the German Clinical Trials Register

Clinical Trial Number: DRKS00005367

Registration date: 10/17/2013

**Supplementary Information:**

The online version contains supplementary material available at https://doi.org/10.1186/s12889-026-26732-7.

## Introduction

Both psychosocial and biological factors decisively affect the susceptibility, progression, and outcomes of infectious diseases. During the pandemic caused by severe acute respiratory syndrome coronavirus 2 (SARS-CoV-2), for example, transmission rates were highest in precarious and overcrowded housing conditions. Furthermore, incidence and mortality were disproportionately elevated among socioeconomically disadvantaged and socially marginalized groups, with men facing a greater risk of severe outcomes and mortality compared to women within these populations [1–4]. Further relevant risk factors identified include age, obesity, and multimorbidity [5, 6]. Notably, similar patterns are also observed in other infectious diseases [7–10]. This potpourri of aggravating factors strongly supports the interplay between psychosocial and biological determinants within different population subgroups.

The influence of sex (biological) and gender (socially constructed) on respiratory infectious diseases has been described previously, although their roles have yet to be fully explored [11, 12]. For example, women are more commonly affected by mild upper respiratory tract infections (URTIs) such as sinusitis, tonsillitis, and otitis, while men tend to experience more severe lower respiratory tract infections (LRTIs), including bacterial and viral pneumonia [13–17]. For example, in seasonal influenza, hospitalization rates are higher in males than in females across all age groups, with mortality rates peaking among children and the elderly [18–20]. Notably, data from Denmark reveal that the risk of hospitalization changes at puberty, with women of reproductive age being at higher risk [21]. In cases of pandemic influenza and outbreaks, disease severity tends to be greater in women of reproductive age compared to their male counterparts [22, 23]. For instance, during the H1N1 pandemic in 2009, Kumar et al. reported that the majority of critically ill patients were young women, a finding echoed by other researchers [22, 24, 25]. Several factors may contribute to these findings. The social construct of gender often assigns specific roles to women and men; for instance, women being more likely to take on caregiving responsibilities that involve extended contact with children or elderly people, spend more time at home, or work in health-related and care-taking fields, which leads to differing exposure patterns to pathogens. Moreover, women experience higher levels of psychosocial stress, increased health awareness, and greater utilization of the healthcare system are reported among women. Additionally, there are notable sex differences in genetic and epigenetic factors, as well as in steroid hormone levels, which affect comorbidities and immune responses [12, 24, 26–28].

The human immune response consists of two components: the innate and adaptive immune responses. Both components are essential for combating pathogens causative for infectious diseases. These immune processes are influenced by multiple factors, including biological sex, age, reproductive status, and psychosocial stress [27, 29]. Immunosenescence impairs both innate and adaptive immune function, leading to increased susceptibility to infections, reduced vaccine efficacy, and a higher risk of severe disease outcomes in older adults [30, 31]. Chronic psychological or social stress can further accelerate immunosenescence [32]. The combined effects of immunosenescence and stress contribute to poorer infection outcomes, including increased morbidity, mortality, and delayed recovery from infectious insults [30, 32–35].

Notably, adult premenopausal females tend to exhibit more robust innate and adaptive immune responses compared to males [36, 37].

Stress, a key mediator of health disparities, affects men and women differently, with women generally experiencing and/ or reporting more stress [38, 39]. Stress can be defined as a threat to an individual’s integrity, which leads to a temporary adaptive process in the individual by triggering the release of stress-mediators, such as cortisol and catecholamines, through the activation of the hypothalamic-pituitary-adrenal axis (HPA) and the sympathetic axis (SA), increasing physiological responses necessary for the ‘fight or flight’ response [40–43]. Both chronic stress and insufficient recovery contribute to overstimulation and exhaustion of the stress response, which can result in long-term health issues such as hypertension, diabetes, depression, and increased susceptibility to infections [44, 45]. Cohen et al. first explored the relationship between stress and URTIs [46]. In several viral challenge studies, higher levels of stress were associated with an increased likelihood of developing URTIs. Notably, individuals with higher PSS scores reported more severe symptoms and greater mucus production. Key stressors identified included chronic stress (≥ 1month), social conflicts, and job insecurity [47–50]. While numerous studies over the few past decades have confirmed the link between perceived stress and the occurrence of URTIs, these analyses often lack gender disaggregation and do not thoroughly examine the complex interplay between sex, gender, stress, and URTIs [51–55]. To address this gap, we conducted a post-hoc analysis of two prospectively recruited study cohorts to test the hypotheses that women experience higher stress levels than men, resulting in increased susceptibility to community-acquired respiratory viruses (CARV) and a greater burden of symptoms. We thereby explored how this might depend on age and pre-infection health by examining different age groups and performing the analysis in both a healthy cohort and a cancer cohort to understand whether stress and URTI patterns differ significantly between these populations.

## Materials and methods

### Study Design and Population

This post hoc analysis utilized data from a monocentric, prospective study conducted at the University Hospital Jena, Germany. A total of 467 individuals were prospectively enrolled between November 2013 and May 2017, all during periods of CARV circulation. The study population comprised 273 healthy individuals (cohort H), primarily students and researchers at the University of Jena, and 194 patients who had undergone hematopoietic stem cell transplantation for cancer treatment at the University Hospital Jena (cohort P) and had thus an impaired immune system function. Eligible participants were aged 18 years or older. Patients were recruited during routine clinical visits, while healthy participants were recruited from the university community. All participants provided written informed consent.

Each cohort completed at least three study visits within a 28-day period. At enrollment, participants underwent a structured interview capturing demographics, household composition (number of people and children), smoking status, and, for patients, cancer-specific features (immunosuppression, graft-versus-host disease, days since transplantation). Biological sex was recorded; gender identity was not assessed, and analyses assumed alignment between sex and gender. The terms “male/men” and “female/women” were used interchangeably. Perceived stress was measured using the Perceived Stress Scale-4 (PSS-4), a validated instrument with established psychometric properties. The PSS-4 was administered in paper format at each visit, and internal consistency was evaluated using Cronbach’s alpha. Symptom burden related to URTI was assessed at each visit using study-specific questionnaires (Baseline Questionnaire, Respiratory Infection Symptom Questionnaire), which have not undergone formal external validation. An English version of these instruments is available in the supplement. Data were entered into a secure electronic database and verified for completeness and accuracy by trained staff. Missing data were managed using complete case analysis, appropriate for low and random missingness in observational studies. All variables were defined a priori, and coding schemes were specified (e.g., sex: male = 0, female = 1; smoking status: current smoker = 1, non-smoker = 0).

Additionally, upper respiratory tract specimens were collected and tested for respiratory viruses by multiplex PCR. Data on epidemiology were published by Rachow et al. [56]. The current analysis focuses solely on the first visit. Due to significant differences between them, the characteristics of cohorts H and P were analyzed separately.

### Measures

Perceived stress was measured using the German version of the PSS-4, which consists of four general questions about feelings and thoughts. The score was originally developed by Cohen et al. and quantifies the extent to which respondents felt their lives were unpredictable, uncontrollable, and overwhelming over the past month [57]. The PSS-4 includes the following items: (1) How often have you felt that you were unable to control the important things in your life? (2) How often have you felt confident about your ability to handle your personal problems? (3) How often have you felt that things were going your way? (4) How often have you felt that difficulties were piling up so high that you could not overcome them [57]? . In accordance with Herrero et al., responses were scored on a five-point scale, ranging from never [1] to very often [5, 58]. After reversing the scores for the two positively worded items (Items 2 and 3), a total PSS-4 score was calculated by summing all four items. The internal consistency in the current study was Cronbach alpha = 0.78.

The study was approved by the local ethics committee (Nr. 3891-09/13) and is registered at German Clinical Trials Register (DRKS00005367).

### Statistical analysis

Categorical variables were described as counts and percentages. Continuous variables were presented as means with standard deviation (± SD) or as medians with interquartile ranges (IQR), depending on distributional characteristics. Where appropriate, group comparisons for categorical variables were performed using Chi-square or Fisher’s exact tests, and associations between categorical variables were quantified using the Phi coefficient (Φ). Comparisons of continuous data between groups were conducted using two-sided t-tests, in accordance with recommendations that mean (M) are informative even for non-normally distributed data. Internal reliability of the PSS-4 was assessed using Cronbach’s alpha, consistent with psychometric standards in HSCT research.

Pearson’s correlation coefficient was used to examine linear associations between PSS-4 and continuous variables such as age and symptom burden (number of symptoms) [59, 60] [1–2]. Scatterplots stratified by sex, with separate linear regression lines for men and women, were used to visualize patterns of association.

Effect sizes were interpreted according to Cohen’s benchmarks: small (d ≥ 0.2; η² = 0.01), medium (d ≥ 0.5; η² = 0.06), and large (d ≥ 0.8; η² = 0.14) [61]. All statistical tests were two-sided, and statistical significance was set at *p* < 0.05 [59]. Estimates, confidence intervals, and p-values were reported in accordance with current statistical review standards [59]. The analyses were performed using SPSS version 26 (IBM Inc., Armonk, NY).

## Results

### Cohort H

The first cohort comprised 273 healthy individuals. An equal number of women and men were included, with the majority being under 30 years of age (Table 1). In both women and men, the number of individuals living alone was comparable, while women were more likely to have children compared to men (12% vs. 7.5%, *p* = 0.302), with most children being 10 years of age or younger. A slightly higher proportion of men than women reported regular smoking. Additionally, the average number of cigarettes smoked per day was nearly twice as high among male participants compared to their female counterparts (*M* = 11.65 (*SD* = 7.31) vs. *M* = 6.95 (*SD* = 5.42), *p* = 0.068).

**Table 1:** Characteristics of the study samples, cohort *H* Healthy individuals

*cohort H*	Women, *n*/*N* (%) 137/273 (50)	Men, *n*/*N* (%) 136/273 (50)	*p*-value
*Age*			
Median (IQR)	24 (20–29)	27 (22.25–36.75)	0.006
≤ 30, n/N (%)	108/137 (79)	90/136 (66)	0.198
31–40, n/N (%)	9/137 (6.5)	15/136 (11)	
41–50, n/N (%)	6/137 (4.5)	12/136 (9)	
51–60, n/N (%)	8/137 (6)	12/136 (9)	
> 60, n/N (%)	6/137 (4.5)	7/136 (5)	
*Housing / living situation*			
Living alone, n/N (%)	27/137 (19.5)	24/136 (17.5)	0.756
Children, n/N (%)	16/135 (12)	10/134 (7.5)	0.302
Number of children, mean (+/-SD)	1.38 (+/-0.62)	1.5 (+/-0.53)	0.602
Smoking, n/N (%)	14/137 (10)	23/136 (17)	0.155
Number of cigarettes/ day, mean (+/-SD)	6.95 (+/-5.42)	11.65 (+/-7.31)	0.068

*SD* Standard deviation,*IQR *Interquartile range

At the time of sampling, the overall occurrence of symptoms did not differ significantly between sexes; however, further analysis revealed that women were significantly more likely to experience moderate and/or severe symptoms (Table 2, suppl. Figure 1a). Specifically, 57% of female participants and 36% of male participants reported experiencing moderate to severe symptoms, while 43.5% of females and 26.5% of males reported severe symptoms. The association between sex and severity of symptoms was weak (φ = 0.210, *p* = 0.181). Common symptoms included cough, sore throat, cold, and headache. Among all healthy participants, fewer than 10% tested positive for CARV-infection via PCR.

**Table 2:** Number and severity of URTI symptoms in the study samples at the time of sampling, cohort H: healthy individuals

*Cohort H*	Women, *n*/*N* (%) 137/273 (50)	Men, *n*/*N* (%) 136/273 (50)	*p*-value	Effect size
*Symptoms at time of sampling*				
Symptoms at time of sampling, n/N (%)	113/137 (82.5)	106/136 (78)	0.366	
No. of symptoms, Mean (+/- SD)	2.45 (+/- 1.67)	2.04 (+/- 1.65)	0.074	
Moderate to severe symptoms, n/N (%)	78/137 (57)	49/136 (36)	< 0.001	φ 0.210
No. of moderate to severe symptoms, Mean (+/- SD)	0.88 (+/- 1.53)	0.64 (+/- 1.17)	0.142	
Severe symptoms, n/N (%)	60/137 (43.5)	36/136 (26.5)	0.003	φ 0.181
No. of severe symptoms, Mean (+/- SD)	0.29 (+/- 0.71	0.16 (+/- 0.53)	0.088	
PCR confirmed CARV-infection, n/N (%)	8/137 (6)	7/136 (5)	1.000	

*SD *Standard deviation

In comparing the mean PSS-4 between women and men, women scored significantly higher than men [*t*(271) = 2.84, *p* < 0.001, *d* = 0.636]; Cohens’ d indicates a medium to large effect size. Significant differences were observed for each of the four items (suppl. Table 1).

Detailed results on perceived stress among females and males across various age groups and living situations are presented in suppl. Table 2. Notable peaks in perceived stress were observed in women under 30 years [*t*(196) = 2.78, *p* < 0.001, *d* = 0.601] and in those aged 51–60 years [*t* [10] = 3.40, *p* = 0.415, *d* = 0.491], whereas women over 60 exhibited the lowest PSS-4 scores. In men, PSS-4 scores decreased with increasing age, showing a statistically significant inverse correlation (*r* = − 0.214, *p* = 0.013). The lowest PSS-4 scores were observed in men over 60.

**Fig. 1:**
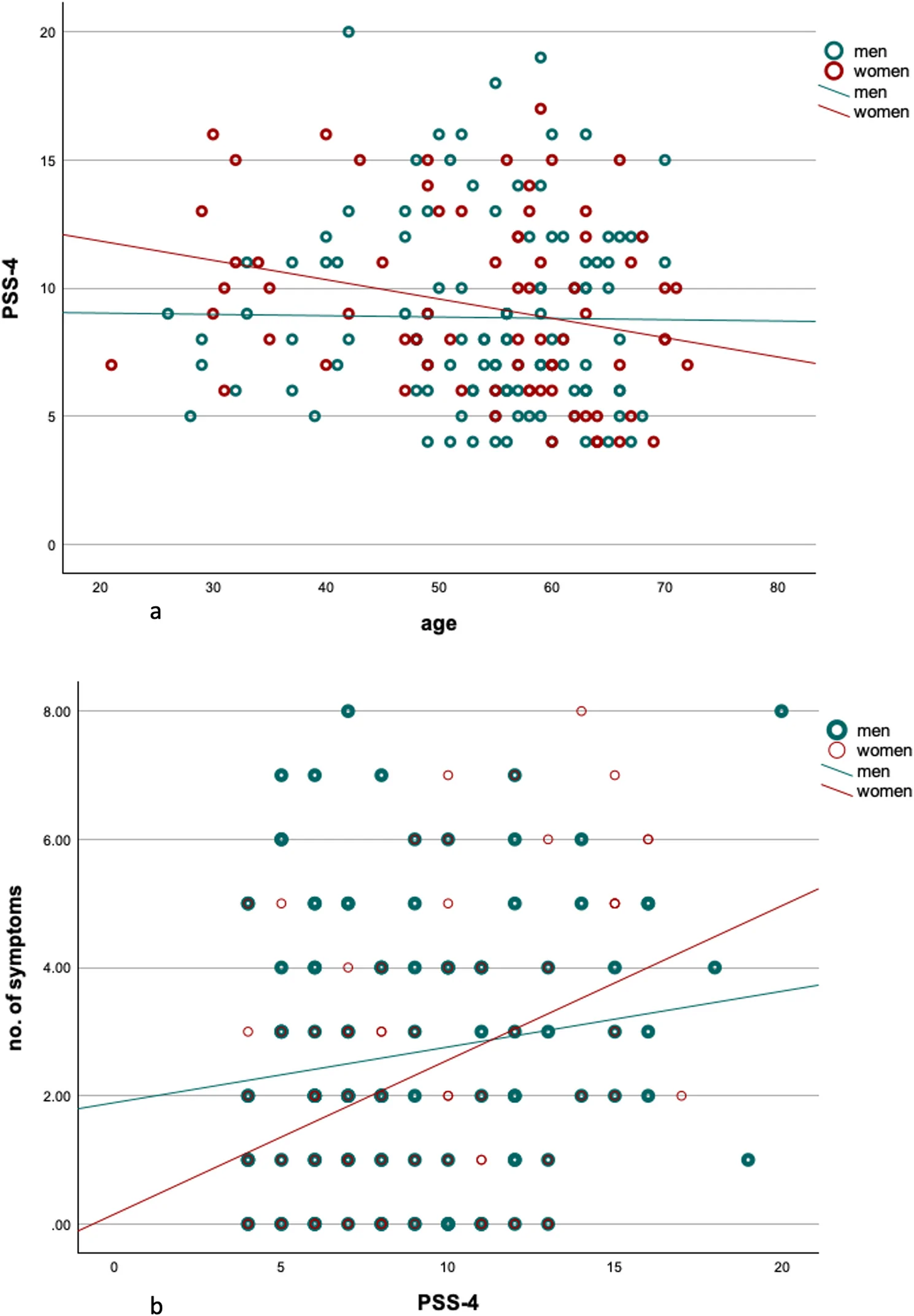
**a** Association between age and PSS-4 in cohort H, stratified by gender. **b** Association between PSS-4 and symptom burden (number of symptoms) in cohort H, stratified by gender. Linear regression lines indicate trends for men and women

Pearson correlations revealed that higher stress levels were modestly associated with female sex (*r* = 0.301, *p* < 0.001) and younger age (*r* = − 0.213, *p* < 0.001). Stress was not significantly associated with living situation, parenthood or smoking status (all |r| < 0.07, all *p* > 0.05). Symptom burden showed no meaningful correlations with perceived stress (*r* = 0.095, *p* = 0.061) and was largely unrelated to sociodemographic variables, with the exception of a very small negative association with age (*r* = − 0.109, *p* = 0.038). Detailed Pearson correlation coefficients and analyses of age, PSS-4, symptom burden, and gender effects are provided in supplementary Tables 3 and Fig. 1.

Bivariate correlations showed small positive associations between stress and symptom burden (*r* = 0.101, *p* = 0.048) and between stress and sex (*r* = 0.109, *p* = 0.037). Stratified analyses revealed that in women, perceived stress was positively associated with symptom burden (*r* = 0.15, *p* = 0.04), whereas in men no significant association was observed (*r* = 0.03, *p* = 0.70). The Stress × Sex interaction in the regression was not statistically significant (β = − 0.097, *p* = 0.215), indicating that sex did not significantly moderate the relationship between stress and symptom burden.

Subsequently, a multiple linear regression was conducted to examine whether gender, age, living situation, having children, and smoking status predicted PSS-4 scores. The model was statistically significant (F(6,260) = 6.06, *p* < 0.001) and explained 12.3% of the variance in PSS-4 scores (adjusted R² = 0.102). Gender and age emerged as significant predictors: females reported higher stress scores than males (B = 1.603, SE = 0.355, β = 0.269, *p* < 0.001), and older participants reported slightly lower stress scores (B = − 0.038, SE = 0.014, β = − 0.161, *p* = 0.009). Living situation, parenthood, smoking status, and symptom burden were not significant predictors (all *p* ≥ 0.405). Multicollinearity was not a concern (tolerance values 0.894–0.981; VIF 1.02–1.12). Standardized residuals ranged from − 2.16 to 3.50, indicating acceptable model behavior.

### Cohort P

The second cohort comprised 194 stem cell recipients with a higher proportion of males (59.5% vs. 40.5%) and an older average age (Table 3). Analysis of patients’ data revealed minimal differences between sexes. Notably, male patients were slightly more likely to have children (31% vs. 24%, *p* = 0.445) and to live alone (19% vs. 10.5%, *p* = 0.136). Again, smoking prevalence was higher among men, although women reported a greater average number of cigarettes smoked per day (*M* = 10.13 (*SD* = 9.2) vs. *M* = 4.78 (*SD* = 5.2), *p* = 0.070). The stem cell specific data indicated comparable characteristics between both sexes. Immunosuppression was observed in 58% of female patients and 63.5% of male patients (*p* = 0.557), while GvHD affected 36.5% of females and 38.5% of males (*p* = 0.881). Most patients participated in the trial more than 100 days after their stem cell transplantation.

**Table 3:** Characteristics of the study samples, cohort *P* Patients with cancer

*cohort P*	Women, *n*/*N* (%) 79/194 (40.5)		Men, *n*/*N* (%) 115/194 (59.5)	*p*-value
*Age*				
Median (IQR)	57 (49–63)		56 (49–62)	0.144
≤ 30, n/N (%)	4/79 (5)		4/115 (3.5)	0.811
31–40, n/N (%)	9/79 (11.5)		9/115 (8)	
41–50, n/N (%)	11/79 (14)		20/115 (17.5)	
51–60, n/N (%)	31/79 (39)		50/115 (43.5)	
> 60, n/N (%)	24/79 (30.5)		32/115 (28)	
*Housing / living situation*				
Living alone, n/N (%)	7/68 (10.5)		19/100 (19)	0.136
Children, n/N (%)	17/71 (24)		33/107 (31)	0.445
Number of children, mean (+/-SD)	1.38 (+/-0.65)		1.31 (+/-0.54)	0.280
Smoking, n/N (%)	8/72 (11)		18/108 (16.5)	0.395
Number of cigarettes/ day, mean (+/-SD)	10.13 (+/-9.2)		4.78 (+/-5.2)	0.070
*Stem cell specific features*				
Immunosuppression, n/N (%)	46/79 (58)		73/115 (63.5)	0.557
GvHD, n/N (%)	29/79 (36.5)		44/115 (38.5)	0.881
Days after SCT, Median (IQR)	648 (100–1,636)		686 (144–2,180)	0.770
≤ 100d, n/N (%)	20/79 (25.5)		22/115 (19)	0.554
> 100d, n/N (%)	59/79 (74.5)	93/115 (81)		

*SD *Standard deviation,* IQR *Interquartile range,* d *days

At the time of sampling, men exhibited a significantly higher likelihood of experiencing URTI symptoms compared to women (68% vs. 83%, *p* = 0.021, φ = -0.177, Table 4). Additionally, although not statistically significant, moderate to severe and severe URTI symptoms were more prevalent among men. The most frequently reported URTI symptoms included cold, myalgia, and headache (suppl. Figure 1b). PCR confirmed CARV-infections were detected in 11.5% and 15% for female and male patients, respectively.

**Table 4:** Number and severity of URTI symptoms in the study samples at the time of sampling, cohort *P* Patients with cancer

*Cohort P*	Women, *n*/*N* (%) 79/194 (40.5)	Men, *n*/*N* (%) 115/194 (59.5)	*p*-value	Effect size
*Symptoms at time of sampling*				
Symptoms at time of sampling, n/N (%)	51/75 (68)	94/113 (83)	0.021	φ -0.177
No. of symptoms, Mean (+/- SD)	2.20 (+/- 2.22)	2.65 (+/- 2.10)	0.155	
Moderate to severe symptoms, n/N (%)	21/79 (26.5)	37/115 (32)	0.188	
No. of moderate to severe symptoms, Mean (+/- SD)	1.47 (+/- 1.99)	1.48 (+/- 1.75)	0.971	
Severe symptoms, n/N (%)	6/79 (7.5)	13/115 (11.5)	0.467	
No. of severe symptoms, Mean (+/- SD)	0.44 (+/-1.01)	0.44 (+/-0.93)	0.998	
PCR confirmed CARV-infection, n/N (%)	9/79 (11.5)	18/115 (15.5)	0.527	

*SD *Standard deviation

Women scored slightly higher on the PSS-4 compared to men [*t*(183) = 3.60, *p* = 0.084, *d* = 0.241] (suppl. Table 4). This trend was evident across all PSS-4 items, particularly for the item regarding the perception of difficulties piling up to an overwhelming extent [*t*(185) = 1.12, *p* = 0.030, *d* = -0.328] (suppl. Table 1). In female patients, the PSS-4 decreased with increasing age (*r* = -0.261, *p* = 0.026). Conversely, male patients exhibited the lowest level of perceived stress at age 30 or younger [*t* [6] = 3.1, *p* = 0.117, *d* = 1.292], while those aged 41 to 50 reported the highest levels [*t* [28] = 3.78, *p* = 0.895, *d* = 0.051].

**Fig. 2:**
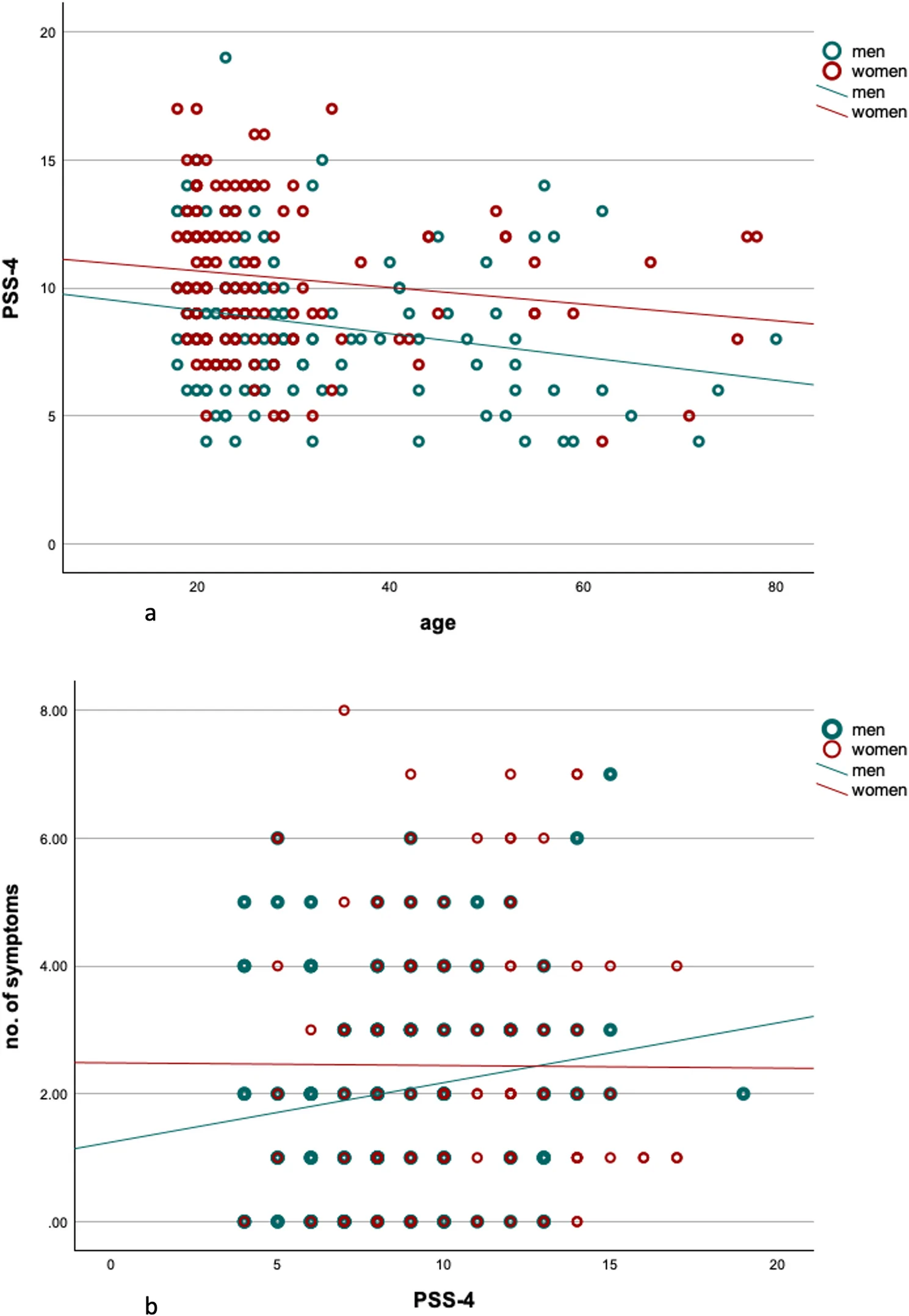
**a** Association between age and PSS-4 in cohort P, stratified by gender. **b** Association between PSS-4 and symptom burden (number of symptoms) in cohort P, stratified by gender. Linear regression lines indicate trends for men and women

Beyond sex and age patterns within subgroups, Pearson correlations showed that burden of symptoms was positively associated with perceived stress (*r* = 0.293, *p* < 0.001). Stratified by sex, this association was stronger in women (*r* = 0.385, *p* < 0.001) than in men (*r* = 0.149, *p* = 0.116). Sex alone was not significantly correlated with symptom burden (*r* = − 0.063, *p* = 0.197). Age demonstrated a modest negative association with stress (*r* = − 0.16, *p* = 0.020). All other sociodemographic variables—including living situation, parenthood, and smoking status—showed no significant bivariate associations with stress (all |r| < 0.14, all *p* > 0.05). Symptom burden was also not significantly correlated with these sociodemographic characteristics. Detailed Pearson correlation coefficients and analyses of age, PSS-4, symptom burden, and gender effects are provided in supplementary Tables 5 and Fig. 2.

A multiple linear regression model including sex, living situation, parenthood, smoking status, age, and symptom burden was conducted to identify independent predictors of perceived stress. The model was statistically significant (F(6,155) = 3.50, *p* = 0.003) and explained 11.9% of the variance in PSS-4 scores (adjusted R² = 0.085). Symptom burden emerged as the only significant independent predictor (B = 0.45, SE = 0.13, β = 0.27, *p* < 0.001), indicating that higher symptom scores were associated with higher levels of perceived stress. Age showed a nonsignificant trend towards lower stress at higher age (B = − 0.04, *p* = 0.106). Sex, living situation, parenthood, and smoking status were not significant predictors in the adjusted model (all *p* > 0.20).

Multicollinearity was not a concern (tolerance > 0.90; VIF < 1.10). Standardized residuals ranged from − 1.80 to 2.69, indicating an acceptable distribution and no major deviations from model assumptions.

## Discussion

The purpose of this post hoc analysis was to evaluate the relationship between perceived stress and the burden of URTIs in both healthy individuals and patients with compromised immune systems due to stem cell transplantation for cancer treatment, with a particular focus on gender disparities. We proposed two hypotheses: first, that women experience higher levels of perceived stress than men; and second, that the PSS-4 correlates with the occurrence and severity of URTIs, resulting in a greater burden of URTIs in distressed individuals. To address these hypothesizes, our study population comprised two distinct cohorts. The first cohort consisted of healthy individuals predominantly aged 20 to 30, with a balanced sex ratio and comparable sociodemographic profiles. In this group, women reported higher stress levels than men, and younger age was associated with greater stress; however, the correlation between PSS-4 scores and URTI burden was weak and not statistically significant, and gender did not significantly moderate this relationship. The second cohort differed markedly and included patients aged 50 to 60 with a male-biased sex ratio who had undergone stem cell transplantation. In this immunocompromised group, perceived stress was a significant independent predictor of URTI symptom burden, with a stronger association in women than in men. Here, higher PSS-4 scores were linked to an increased symptom burden, and women showed a steeper rise in symptom burden as stress levels increased. Overall, the impact of stress on infection risk and severity appears amplified in immunocompromised patients, potentially due to stress-mediated immune dysregulation and altered coping mechanisms [60, 62–64].

Consistent with our first hypothesis, women, especially those of reproductive age, consistently reported higher perceived stress than men. In healthy populations, both sex and age were significant predictors of stress: younger adults and women show the highest stress levels, with stress decreasing with age for both sexes. In patients with cancer, neither age nor sex significantly predicted stress, although a nonsignificant trend toward lower stress with increasing age was observed.

Numerous global studies support these findings, consistently reporting higher perceived stress in women across age groups, while stress levels generally decline with age and remain elevated in women across diverse sociodemographic variables, such as income, education, race, marital status, and household composition [38, 39, 65].

Recent American Psychological Association data confirm rising stress levels, particularly among women and adults aged 18–44, with the COVID-19 pandemic amplifying these disparities [66, 67]. The survey also suggests that men tend to be less aware of stress and its effects on health, which may contribute to underreporting and a higher long-term risk of chronic disease [68]. In contrast, older adults generally report lower stress levels, likely reflecting different stressors across the lifespan and the adoption of more effective coping strategies with advancing age [66, 69–71]. However, women consistently report higher stress levels than men, driven by a combination of biological factors and gendered social roles, as demonstrated in longitudinal and cross-sectional studies [72, 73].

The COVID-19 pandemic, compounded by global conflicts, racism, inflation, and climate-related disasters, led to increased psychological distress and vulnerability to URTIs in women and younger adults compared to older individuals. These groups experienced higher rates of anxiety, depression, and stress, with these disparities amplified during periods of heightened socioeconomic pressure and public health crises [74–79]. Socioeconomic disadvantage, including lower income and unemployment, further increased risk, especially among women and young people [78–80].

Biological sex differences also contribute to these patterns. Women exhibit distinct neuroendocrine profiles, including blunted cortisol responses and higher DHEA levels as well as estrogen modulated innate and adaptive immunity [73, 81]. These mechanisms heighten vulnerability to stress-related immune dysregulation and internalizing disorders, particularly during reproductive years [73, 74, 82]. Gendered social roles and socioeconomic pressures further intensify stress perception, with loneliness emerging as a key mediator of adverse mental health outcomes in women during the pandemic [72, 83, 84]. Our study, however, did not collect detailed data on these psychosocial dimensions, highlighting a need for future research to explore why stress levels fluctuate throughout a lifetime and how these factors interact with biological determinants in shaping health outcomes in men and women.

As mentioned earlier, stress levels declined with age, particularly among healthy individuals. In the patient cohort, age-related patterns were inconsistent: female patients showed a modest age-related decrease in stress, while male patients exhibited a non-significant U-shaped pattern, with higher stress in middle age. These trends did not reach statistical significance, and in the linear regression model neither age nor sex predicted stress; instead, symptom burden remained the only significant independent predictor. Interestingly, male patients over 60 reported experiencing higher stress levels than their female counterparts.

This observation may reflect changing caregiving roles, the psychological burden of chronic illness, the persistence of work-related stress despite declining physiological reserves, or age-related testosterone decline. Changing caregiving roles are increasingly common as men age, with many assuming responsibilities for spouses or family members with chronic illness [85]. Caregiving is a well-established source of chronic stress, leading to dysregulation of the HPA axis, increased cortisol, and impaired immune function, particularly in elderly caregivers [86–89].

The psychological burden of chronic illness is a major contributor to stress in older adults [90–92]. However, sex-disaggregated data on perceived stress in cancer patients remain limited. Although cancer diagnosis and treatment introduce substantial stressors, perceived stress typically decreases with effective therapy, improved disease control, and the passage of time [93, 94]. In our cohort, most patients completed the survey more than 100 days after transplantation, suggesting some degree of adaptation; however, one-third reported GvHD, which may represent a persistent stressor alongside concerns about relapse. Disease activity and remission status were not assessed.

Persistence of work-related stress despite declining physiological reserves is another important factor. Older adults who remain in the workforce, especially in physically demanding or low-control jobs, experience higher stress and depressive symptom [95–97]. Poor psychosocial work environments and lack of organizational support are consistently linked to elevated stress scores in senior workers [96, 97].

Age-related declines in testosterone and functional hypogonadism are common in middle-aged and older men, even with an intact hypothalamic-pituitary-gonadal axis [98–100]. Lower testosterone is associated with increased frailty, reduced stress resilience, and higher risk of adverse health outcomes, including cardiovascular disease and mortality [100–102].Testosterone modulates the HPA axis and stress reactivity; its decline may contribute to heightened stress responses and diminished capacity to cope with chronic stressors [100, 101, 103]. In addition, hypogonadism may increase infection susceptibility through elevated pro-inflammatory cytokines and altered immune function [104]. In contrast, estradiol enhances HPA axis activity and contributes to generally higher stress responsivity in females. This effect is most pronounced during periods of high endogenous estradiol, such as the follicular phase, and underpins sex differences in stress responsivity, with females generally exhibiting greater HPA axis activation than males [105–109]. Its decline during menopause is associated with reduced HPA activation, whereas estradiol therapy in postmenopausal women has been shown to attenuate stress-induced cortisol release and mitigate stress-related cognitive effects [110–112].

Our second hypothesis was partially supported. In healthy individuals, women exhibited a higher stress and a greater likelihood of moderate to severe URTI symptoms, but perceived stress was not significantly associated with URTI number or severity, with only a non-significant trend suggesting slightly greater symptom burden in younger women. This finding is consistent with population-based and experimental data showing that, while psychological stress is associated with increased URTI risk, the effect size in healthy cohorts is modest and often not statistically significant, especially when controlling for confounders [47, 51, 113]. In contrast, among immunocompromised patients, perceived stress was a significant independent predictor of URTI burden, with higher PSS-4 scores linked to increased symptom burden. This effect was stronger in women, who exhibited a steeper rise in symptom burden with increasing stress. Meta-analytic evidence confirms that psychological stress robustly increases susceptibility to URTI in vulnerable populations, with effect sizes consistent across stress types and assessment methods [50, 51, 114].

Nonetheless, there was a slight trend indicating increased stress levels and a greater burden of URTIs correlate among young adult women. This trend aligns with findings from Groer et al. who reported that premenopausal women experience more symptoms of infectious diseases during their perimenstrual period compared to midcycle, a pattern corresponding with increased levels of perceived stress [115]. However, menstrual cycle phases were not documented in our study, which may confound the relationship between stress and URTI burden. Estrogen and progesterone fluctuations during the menstrual cycle modulate immune function and inflammatory responses, leading to cyclical variation in susceptibility to infection and symptom severity [116–121]. During the luteal phase, higher progesterone, estradiol and cortisol levels are associated with increased regulatory T cell activity and reduced cell-mediated immunity, creating a “window of vulnerability” after ovulation with heightened susceptibility to viral infections [120, 122–127]. Periods of low estradiol, such as perimenstrual or postpartum phases, reduce regulatory T cell numbers and function, increasing baseline inflammation and immune activation [128, 129]. These hormonal effects correspond with epidemiological data showing elevated respiratory symptoms and infection risk in mid-luteal to mid-follicular stages, with premenopausal women experiencing higher morbidity than age-matched men [12–15, 22, 23, 118, 121, 130, 131].

Among patients, perceived stress was positively correlated with URTI symptom burden for both sexes, although overall symptom severity did not differ significantly between men and women. Notably, occurrence of URTI was higher in men. This relationship is robust across multiple prospective studies and is independent of health practices, exposure, and sociodemographic factors [47, 51]. The correlation is mediated by stress-induced immune dysregulation, including increased proinflammatory cytokine production (e.g., IL-6) and altered HPA axis activity, which heighten susceptibility to infection and symptom severity [50, 114, 132]. Although women may exhibit a steeper increase in symptom burden with rising stress, overall symptom severity does not differ significantly between men and women in most clinical cohorts [133, 134]. Sex differences in immune response—such as lower interferon production in males and cyclical hormonal modulation in females—may influence infection risk, but do not consistently translate to differences in symptom severity [133, 134].

Age, treatment history, and overall morbidity are important contributors to infection susceptibility. Older age is associated with impaired immune function and increased risk of URTI [133, 135]. Treatment history, including immunosuppression and stem cell transplantation, further elevates risk by reducing antiviral immunity and increasing vulnerability to severe infection [133]. General morbidity, such as comorbid chronic diseases, also increases susceptibility to URTI and may interact with stress to worsen outcomes [133, 135].

### Limitations

Our analysis has several limitations that may affect the generalizability of the findings. Response and selection biases are possible, with underrepresentation of healthy participants aged ≥ 41 years and patients aged ≤ 50 years. The healthy cohort primarily comprised students or employed individuals, whose life circumstances and stressors may not reflect the general population. The questionnaire did not capture information on gender identity, socioeconomic factors, social support, cultural background, migration status, or detailed clinical data on hematological diseases and comorbidities. Additionally, detailed information on influenza vaccination status and antibiotic treatment was limited, representing a further limitation of this study. Although these variables were assessed, incomplete data—such as timing and indication in relation to the URTI—precluded their inclusion as covariates in the regression models, despite their potential relevance as predictors of disease severity and outcomes.

A longitudinal approach is needed to more comprehensively assess stress dynamics and their interaction with infectious diseases, enabling the identification of potential confounders and monitoring of temporal changes. Future studies should also include direct assessments of sex steroid levels, as unrecognized hormonal variations may confound observed sex differences in stress and immune responses. These considerations are particularly relevant for understanding how menstrual cycle– and pregnancy-related hormonal shifts influence susceptibility to infectious diseases, and how age-related testosterone decline may affect perceived stress. Finally, as our data were collected prior to the COVID-19 pandemic, stress disparities between sexes may be underestimated, given evidence that women experienced disproportionately higher stress during the pandemic [67].

## Conclusion

In summary, our analysis highlights that perceived stress is higher in women, particularly in younger adults, and that stress predicts URTI symptom burden more strongly in immunocompromised patients than in healthy individuals. While sex differences in healthy populations were modest, they became more pronounced under conditions of immune compromise, suggesting an interplay of biological and psychosocial factors. These findings emphasize the importance of considering stress management and psychosocial support, especially in vulnerable populations, to potentially reduce infection severity. Future research should explore longitudinal dynamics of stress, hormonal influences, and social determinants to better understand sex-specific susceptibility to respiratory infections.

## Supplementary Information

Supplementary Material 1.

Supplementary Material 2.

Supplementary Material 3.

Supplementary Material 4.

## Data Availability

The dataset used and analyzed during the current study are available from the corresponding author on reasonable request.

## Abbreviations

CARV: Community–acquired respiratory viruses
COVID: 19–coronavirus disease 2019
GvHD: Graft versus host disease
HPA: Hypothalamic–pituitary–adrenal axis
IQR: Interquartile ranges
LRTI: Lower respiratory tract infection
M: Mean
PSS: Perceived stress scale
SA: Sympathetic axis
SARS: CoV–2
SD: Standard deviation
URTI: Upper respiratory tract infection
