# Supplementary figures and images for: Gender differences and psychosocial stress in upper respiratory tract infections: insights from healthy and hematological cancer cohorts
Source: BMC Public Health. 2026 May 30;26:1784. doi: 10.1186/s12889-026-26732-7 (PMC13231776; doi:10.1186/s12889-026-26732-7)

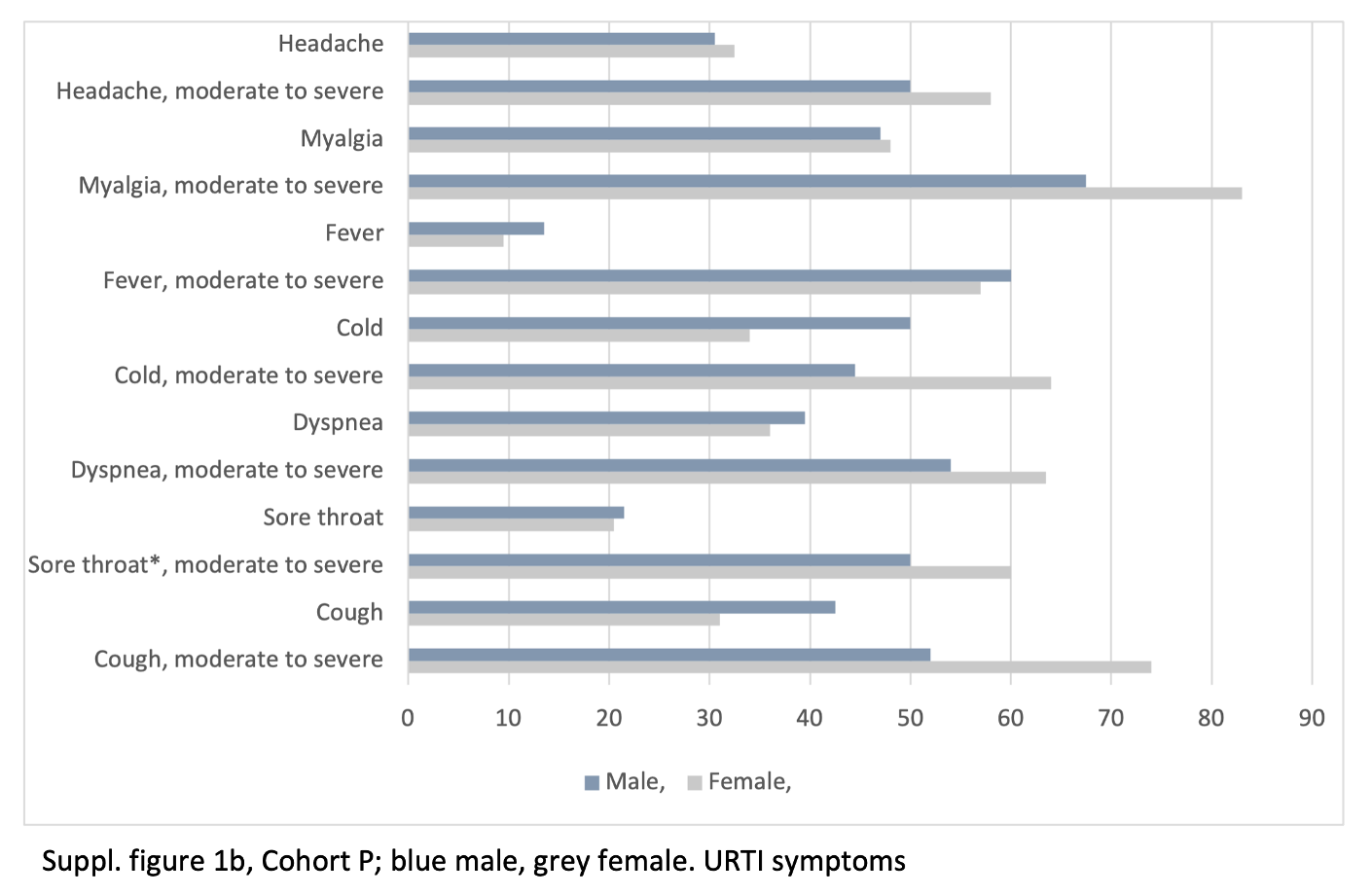

Supplement: Supplementary file 1 — Supplementary Material 1. [file 12889_2026_26732_MOESM1_ESM.png]

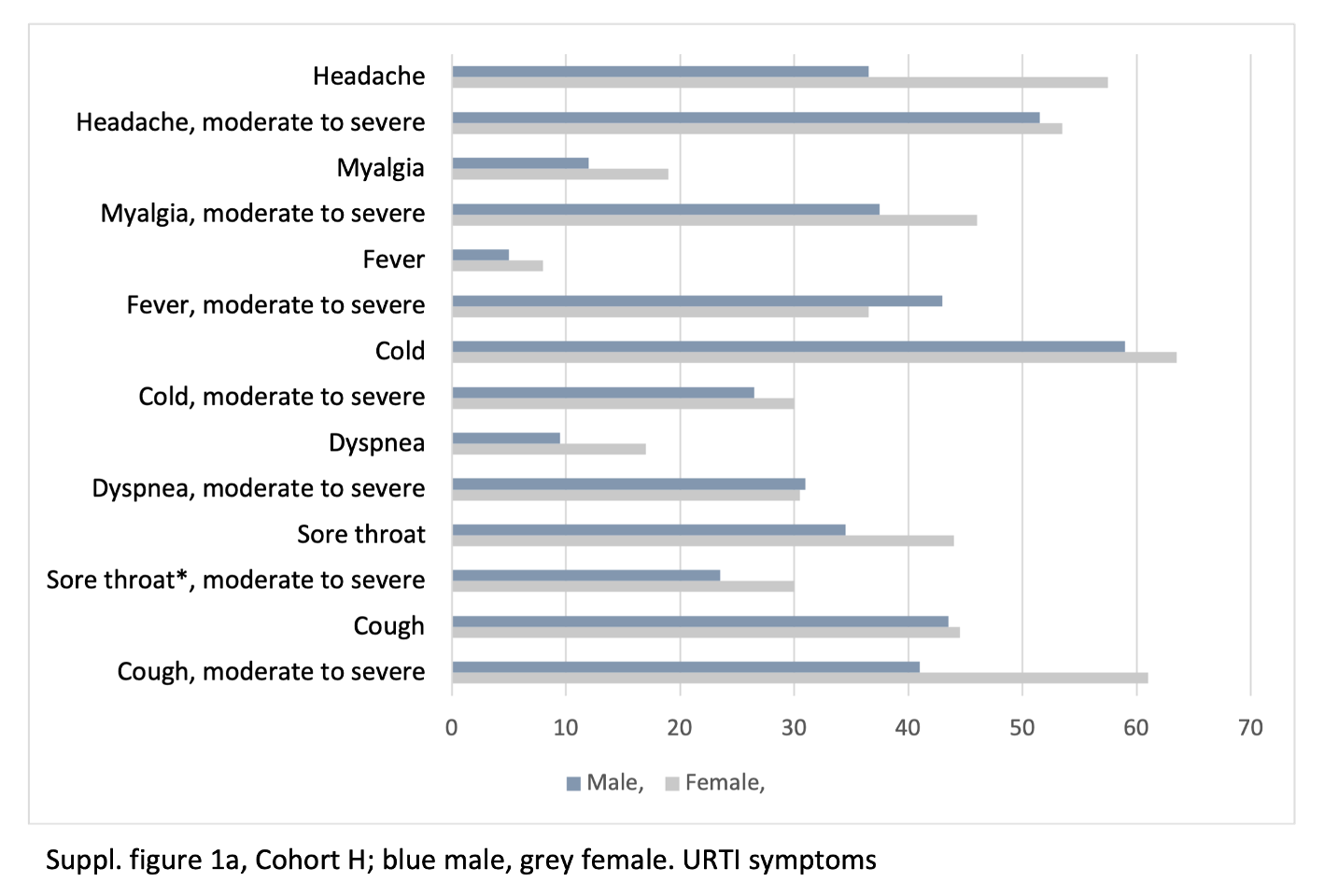

Supplement: Supplementary file 3 — Supplementary Material 3. [file 12889_2026_26732_MOESM3_ESM.png]
