# Supplementary material for: Gender differences and psychosocial stress in upper respiratory tract infections: insights from healthy and hematological cancer cohorts
Source: BMC Public Health. 2026 May 30;26:1784. doi: 10.1186/s12889-026-26732-7 (PMC13231776; doi:10.1186/s12889-026-26732-7)
Supplement: Supplementary file 2 — Supplementary Material 2. [file 12889_2026_26732_MOESM2_ESM.docx]

**Baseline Questionnaire**

Subject number: date:

Sex: Male  Female

Age: ____

Anamnesis

I do not have a chronic respiratory condition (including asthma – even mild forms)

I do not have any specific immunosuppressive conditions (including diabetes mellitus)

For individuals who underwent stem cell transplantation

Days since transplantation: ____

I do not have Graft versus Host disease (GvHD).

I do not take any immunosuppressive medications.

Do you smoke?  no

yes

if yes, how many cigarettes do you smoke per day: ____

Living conditions

I live alone.

I share my kitchen/ bathroom with ____ individuals.

There are ____ children living in my household, ages: ________

Facebook usage

I am not on Facebook.

I am on Facebook but use it infrequently (less often than daily).

I am on Facebook and use it regularly (usually daily).

I have ____ friends on Facebook.

Flu vaccination

no

yes

if yes, date (month/year) ________

**Respiratory Infection Symptom Questionnaire**

Subject number: date/visit:

Please answer the following questions completely.

1. In the last 3 weeks, have you experienced any of the following symptoms?
   (if already described in the last questionnaire, only describe new or ongoing symptoms)

Cough no  mild  moderate  severe  very severe Cough with phlegm no  mild  moderate  severe  very severe
Sore throat no  mild  moderate  severe  very severe Shortness of breath no  mild  moderate  severe  very severe Runny nose no  mild  moderate  severe  very severe Fever no  mild  moderate  severe  very severe Muscle aches no  mild  moderate  severe  very severe Headache no  mild  moderate  severe  very severe

Answer questions 2 – 5 only if you experienced at least one symptom in question 1.

2. Do you still have any of these symptoms? Yes  No

3. Have you been in contact with anyone with similar symptoms, do you suspect an infection? Yes  No

4. Did you visit a doctor because of these symptoms? Yes  No

5. Where you given sick leave from work or your studies due to these symptoms? Yes  No

6. Did you take an antiviral or antibiotic medication? Yes  No
