# Supplementary material for: Gender differences and psychosocial stress in upper respiratory tract infections: insights from healthy and hematological cancer cohorts
Source: BMC Public Health. 2026 May 30;26:1784. doi: 10.1186/s12889-026-26732-7 (PMC13231776; doi:10.1186/s12889-026-26732-7)
Supplement: Supplementary file 4 — Supplementary Material 4. [file 12889_2026_26732_MOESM4_ESM.docx]

Suppl. Table 1. Descriptive analysis of PSS-4 items in both cohorts

| Cohort H | Women, n/N (%)  137/273 (50) | Men, n/N (%)  136/173 (50) | p-value | Effect size (d) |
| --- | --- | --- | --- | --- |
| How often have you felt, that you were unable to control the important things in your life (1) | 2.82 (+/- 0.98) | 2.32 (+/-0.95) | < 0.001 | -0.523 |
| How often have you felt confident about your ability to handle your personal problems? (2) | 2.49 (+/- 0.89) | 2.04 (+/- 0.81) | < 0.001 | -0.535 |
| How often have you felt that things were going your way? (3) | 2.68 (0.87) | 2.25 (0.89) | < 0.001 | -0.484 |
| How often have you felt difficulties were piling up so high that you could not have overcome them? (4) | 2.41 (+/- 1.06) | 1.99 (+/- 1.04) | 0.001 | -0.400 |
| Cohort P | **Women, n/N (%)**  **79/194 (40.5)** | **Men, n/N (%)**  **115/194 (59.5)** | **p-value** | **Effect size (d)** |
| How often have you felt, that you were unable to control the important things in your life (1) | 2.68 (+/- 1.27) | 2.38 (+/-1,22) | 0.107 | -0.243 |
| How often have you felt confident about your ability to handle your personal problems? (2) | 2.16 (+/- 1.02) | 2.1 (+/-1.16) | 0.701 | -0.058 |
| How often have you felt that things were going your way? (3) | 2.57 (+/- 0.97) | 2.43 (+/-1.15) | 0.392 | -0.128 |
| How often have you felt difficulties were piling up so high that you could not have overcome them? (4) | 2.19 (+/- 1.18) | 1.82 (+/- 1.08) | 0.030 | -0.328 |

Suppl. Table 2: Perceived stress (means and standard deviations of PSS-4 scores) in the study samples, cohort H: healthy individuals.

| cohort H | Women,  n/N (%) 137/273 (50) | Men,  n/N (%) 136/273 (50) | p-value | *Effect size* |
| --- | --- | --- | --- | --- |
| All healthy individuals | 10.4 (+/-2.78) | 8.6 (+/- 2.89) | <0.001 | d 0.636 |
| Age |  |  | 0.020 | 0.020 |
| ≤ 30, (n= 198) | 10.59 (+/-2.74) | 8.92 (+/- 2.83) | < 0.001 | d 0.662 |
| 31 – 40, (n= 24) | 9.78 (+/- 3.63) | 8.4 (+/- 2.92) | 0.318 | d 0.431 |
| 41 – 50, (n= 18) | 9.33 (+/- 2.16) | 8.25 (+/-2.41) | 0.368 | d 0.463 |
| 51 – 60, (n=20) | 10.71 (+/- 1.7) | 7.58 (+/- 3.48) | 0.041 | d 1.053 |
| > 60, (n= 13) | 8.67 (+/- 3.56) | 7.0 (+/- 3-22) | 0.415 | d 0.491 |
| Housing / living situation |  |  |  |  |
| Living alone |  |  | 0.727 |  |
| Yes, (n= 51) | 10.33 (+/-2.77) | 8.6 (+/-2.83) |  |  |
| No, (n=222) | 10.7 (+/-2.87) | 8.58 (+/- 3.23) |  |  |
| Children |  |  | 0.864 |  |
| Yes, (n= 26) | 10.13 (+/- 2.80) | 8.0 (+/- 2.40) |  |  |
| No, (n=243) | 10.46 (+/- 2.8) | 8.67 (+/-2.94) |  |  |
| Smoking |  |  | 0.244 |  |
| Yes, (n= 37) | 10.43 (+/- 3.01 | 8.13 (+/- 2.65) |  |  |
| No, (n=236) | 10.4 (+/-2.77) | 8.7 (+/-2.94) |  |  |
| *SD – standard deviation* | | |  |  |

| **Suppl. Table 3:** Summary of age, stress (PSS-4), symptom burden, and gender effects in cohort H: healthy individuals. | | | | | | | | |
| --- | --- | --- | --- | --- | --- | --- | --- | --- |
| Analysis | **Variable 1** | **Variable 2 / Predictor** | **r / B (unstandardized)** | **β (standardized)** | **SE** | **t** | **p**-value | **95% CI** |
| Pearson correlations | Stressscore (PSS-4) | Sex | 0.304 | – | – | – | <.001 | – |
|  | Stressscore (PSS-4) | Age | –0.213 | – | – | – | <.001 | – |
|  | Stressscore (PSS-4) | Living situation | –0.047 | – | – | – | 0.220 | – |
|  | Stressscore (PSS-4) | Parenthood | –0.024 | – | – | – | 0.349 | – |
|  | Stressscore (PSS-4) | Smoking status | –0.066 | – | – | – | 0.142 | – |
|  | Stressscore (PSS-4) | Symptom burden | 0.101 | – | – | – | 0.048 | – |
| Stratified correlations | Women | Stress – Symptom burden | 0.15 | – | – | – | 0.04 | – |
|  | Men | Stress – Symptom burden | 0.03 | – | – | – | 0.70 | – |
| Multiple linear regression | Constant | – | 8.212 | – | 0.852 | 9.640 | <.001 | 6.535 – 9.890 |
|  | Sex (female) | – | 1.603 | 0.269 | 0.355 | 4.511 | <.001 | 0.903 – 2.302 |
|  | Age | – | –0.038 | –0.161 | 0.014 | 2.628 | 0.009 | –0.066 – 0.009 |
|  | Living situation | – | –0.178 | –0.023 | 0.461 | 0.387 | 0.699 | –1.086 – 0.729 |
|  | Parenthood | – | –0.051 | –0.005 | 0.612 | 0.083 | 0.934 | –1.257 – 1.155 |
|  | Smoking status | – | –0.163 | –0.019 | 0.511 | 0.319 | 0.750 | –1.170 – 0.844 |
|  | Symptom burden | – | 0.081 | 0.049 | 0.096 | 0.835 | 0.405 | –0.109 – 0.270 |
| Model Model summary: R² = 0.119, Adjusted R² = 0.085 / F(6,155) = 3.50, p = 0.003 / Multicollinearity: Tolerance = 0.923–0.963, VIF = 1.038–1.101 / Standardized residuals: –1.799 to 2.689 | | | | | | | | |
| *r - Pearson correlation coefficient; B (unstandardized) - unstandardized regression coefficient, β (standardized) - Standardized regression coefficient;SE - Standard error of the estimate or coefficient; t - t-value from the t-test; 95% CI - 95% confidence interval;* | | | | | | | | |

Suppl. Table 4. Perceived stress (means and standard deviations of PSS-4 scores) in the study samples, cohort P: patients with cancer.

| Cohort P | Women, n/N(%)  79/194 (40.5) | Men, n/N (%)  115/194 (59.5) | p-value | *Effect size* |
| --- | --- | --- | --- | --- |
| All patients | 9.27 (+/- 3.56) | 8.85 (+/- 3.64) | 0.084 | d 0.241 |
| Age |  |  | 0.023 | 0.033 |
| ≤ 30, (n= 8) | 11.25 (+/-4.03) | 7.25 (+/- 1.71) | 0.117 | d 1.292 |
| 31 – 40, (n= 18) | 10.44 (+/-3.35) | 8.78 (+/- 2.63) | 0.259 | d 0.552 |
| 41 – 50, (n= 31) | 10.45 (+/- 3.3) | 10.26 (+/-4.03) | 0.895 | d 0.051 |
| 51 – 60, (n= 81) | 9.35 (+/- 3.62) | 8.57 (+/-3.88) | 0.403 | d 0.203 |
| > 60, (n= 56) | 7.83 (+/-3.34) | 8.65 (+/-3.33) | 0.376 | d -0.246 |
| Housing / living situation |  |  |  |  |
| Living alone |  |  | 0.182 |  |
| Yes, (n= 26) | 8.57 (+/- 2.57) | 8.42 (+/- 2-54) |  |  |
| No, (n= 142) | 9.55 (+/-3.74) | 8.88 (+/- 3.57) |  |  |
| Children |  |  | 0.702 |  |
| Yes, (n= 50) | 9.75 (+/-4.04) | 8.75 (+/- 4.27) |  |  |
| No, (n= 128) | 9.17 (+/-3.5) | 8.89 (+/- 3.36) |  |  |
| Smoking |  |  | 0.179 |  |
| Yes, (n= 26) | 10.13 (+/-3.8) | 10.17 (+/-5.04) |  |  |
| No, (n= 154) | 9.33 (+/-3.59) | 8.71 (+/-3.29) |  |  |
| *SD – standard deviation* | | |  |  |

| **Suppl. Table 5:** Summary of age, stress (PSS-4), symptom burden, and gender effects in cohort P: patients with cancer. | | | | | | | | |
| --- | --- | --- | --- | --- | --- | --- | --- | --- |
| Analysis | **Variable 1** | **Variable 2 / Predictor** | **r / B (unstandardized)** | **β (standardized)** | **SE** | **t** | **p**-value | **95% CI** |
| Pearson correlations | Stressscore (PSS-4) | Sex | 0.085 | – | – | – | 0.142 | – |
|  | Stressscore (PSS-4) | Living situation | 0.085 | – | – | – | 0.142 | – |
|  | Stressscore (PSS-4) | Parenthood | 0.012 | – | – | – | 0.440 | – |
|  | Stressscore (PSS-4) | Smoking status | 0.095 | – | – | – | 0.114 | – |
|  | Stressscore (PSS-4) | Age | –0.162 | – | – | – | 0.020 | – |
|  | Stressscore (PSS-4) | Symptom burden | 0.293 | – | – | – | <0.001 | – |
| Stratified correlations | Women | Stress – Symptom burden | 0.385 | – | – | – | <0.001 | – |
|  | Men | Stress – Symptom burden | 0.149 | – | – | – | 0.116 | – |
| Multiple linear regression | Constant | – | 8.598 | – | 1.782 | 4.825 | <0.001 | 5.078–12.118 |
|  | Sex (female) | – | 0.699 | 0.095 | 0.564 | 1.240 | 0.217 | –0.415–1.813 |
|  | Living situation | – | 0.689 | 0.069 | 0.786 | 0.877 | 0.382 | –0.863–2.241 |
|  | Parenthood |  | –0.108 | –0.013 | 0.677 | 0.160 | 0.873 | –1.446–1.230 |
|  | Smoking status | – | 0.909 | 0.076 | 0.921 | 0.987 | 0.325 | –0.911–2.729 |
|  | Age | – | –0.043 | –0.126 | 0.026 | 1.628 | 0.106 | –0.095–0.009 |
|  | Symptom burden | – | 0.449 | 0.266 | 0.131 | 3.434 | <0.001 | 0.191–0.707 |
| Model summary: R² = 0.123, Adjusted R² = 0.102 / F(6,260) = 6.06, p < 0.001 / Multicollinearity: Tolerance = 0.894–0.981, VIF = 1.02–1.12 / Standardized residuals: –2.16 to 3.50, Cook`s distance 0.05 | | | | | | | | |
| *r - Pearson correlation coefficient; B (unstandardized) - unstandardized regression coefficient, β (standardized) - Standardized regression coefficient;SE - Standard error of the estimate or coefficient; t - t-value from the t-test; 95% CI - 95% confidence interval;* | | | | | | | | |
